# Cardiac and respiratory self-gated 4D multi-phase steady-state imaging with ferumoxytol contrast (MUSIC)

**DOI:** 10.1186/1532-429X-18-S1-O51

**Published:** 2016-01-27

**Authors:** Fei Han, Ziwu Zhou, Eric S Han, J Paul Finn, Peng Hu

**Affiliations:** 1 Radiology, University of California, Los Angeles, Los Angeles, CA USA; 2 Harvard Westlake School, Los Angeles, CA USA

## Background

Our recently proposed MUSIC approach acquires ferumoxytol-enhanced 4D images using ECG and airway pressure signal for motion gating. It has been routinely used in our institution and provides detailed information on vascular anatomy. However, the airway pressure signal is only available in patients with general anesthesia (GA) and ECG is often problematic in high field strength. Therefore, we propose the self-gated MUSIC (SG-MUSIC) where cardiac and respiratory motion is compensated by retrospective data sorting base on derived SG signals. In this study, we tested SG-MUSIC on pediatric patients under GA so that the derived SG signal can be validated against the recorded physiological signal and the images compared with the original MUSIC.

## Methods

**Figure 1:**
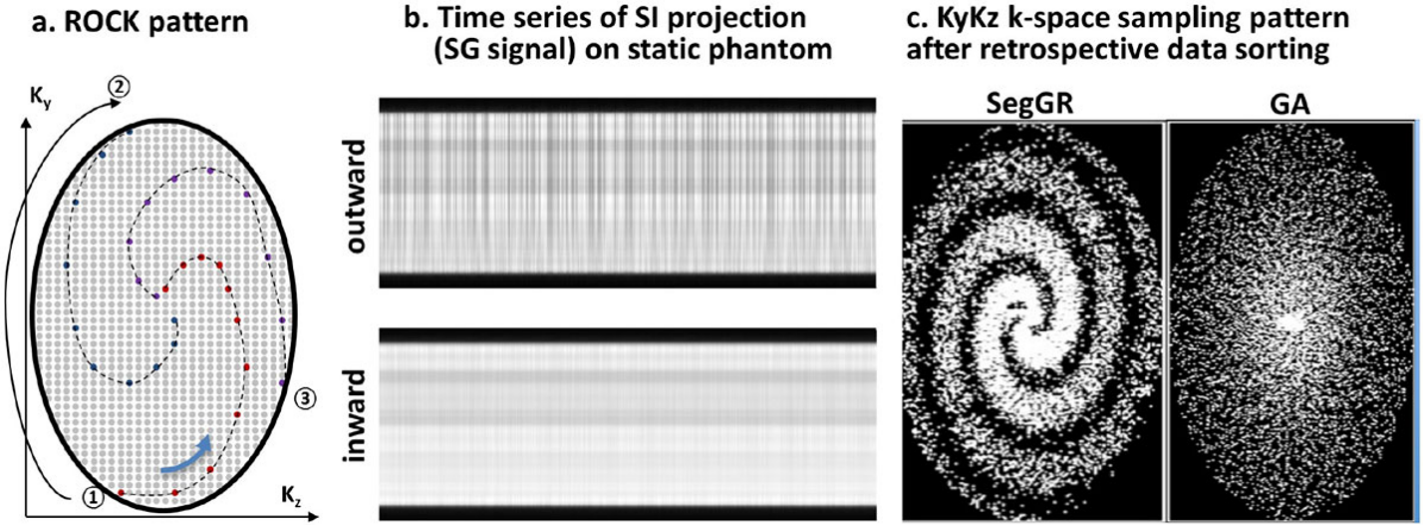
**In the ROtating Cartesian Kspace pattern (a), kykz views of 3D Cartesian grid are reordered in a quasi-spiral manner with each arm starts from the outer, ends at the center kspace and rotates using segmented golden angle [Han et al, MRM2015]**. The time series of SI projection from SG signal on static phantom (b) shows that the inward quasi-spiral arm could significantly reduce the eddy-current interference on self-gating signal because an adjacent kspace line is acquired in previous TR instead of a huge kspace jump in the case of outward arm. The kykz sampling pattern of segGA, due to the extra degree of randomness, is much uniform than that of GA (c), both after a retrospective data sorting using recorded ECG and respiratory signal in previous patient scan. (segGR: segmented golden ratio; GA: golden angle of 137.5°)

The kspace is sampled using ROtating Cartesian Kspace (ROCK) method (Fig. 1) where k_y_k_z_ views of 3D Cartesian grid were reordered using quasi-spiral pattern with each arm starts from the outer and ends at the center kspace. The arms are rotated using segmented golden ratio in which the entire kspace is divided into 7 angular segments, each segment is sampled using golden ratio and different segments are sampled in a pseudo-random order. The extra degree of randomness ensures uniform kspace sampling even after data sorting. Cardiac and respiratory SG signal was derived from the kspace centerline using cross-correlation and band-pass filter. The data is then retrospectively gated based on respiratory SG signal and binned to 6 phases based on cardiac SG signal, followed by ESPIRiT reconstruction. Static phantom experiment was performed to validate the ROCK pattern in different settings: 1)direction of quasi-spiral arms (inward vs. outward); 2) rotating strategy (segmented golden ratio vs. golden angle). Data was sorted using physiological signal recorded from previous patient scans. 6 pediatric patients underwent GA were included in this study. 4 mg-Fe/kg ferumoxytol was administrated. The SG-MUSIC (0.9/2.9 ms, 25°, 1 mm^3^) was performed after clinical protocol, which includes the original MUSIC. ECG and airway pressure signal were recorded.

## Results

**Figure 2:**
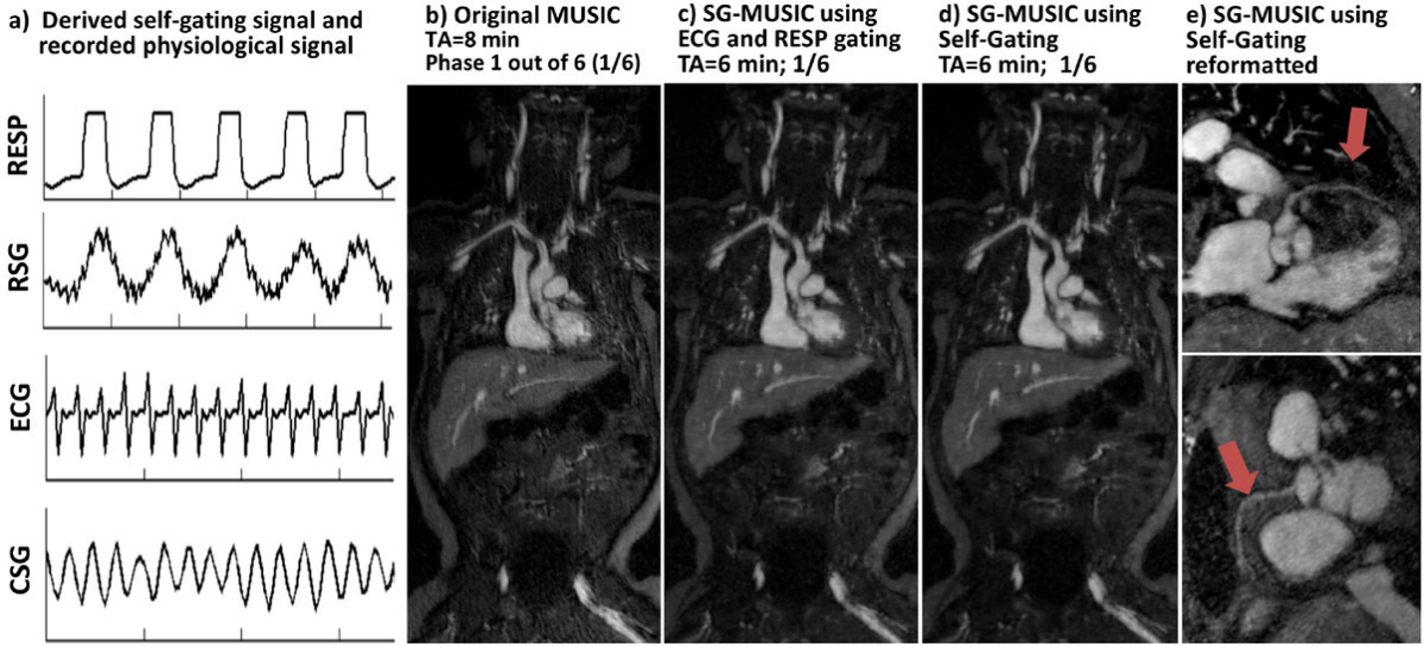
**Data acquired on a 3 month-old female**. Derived SG signal correlates well with the recorded physiological signal (a). The SG-MUSIC reconstructed using SG signal (d) has comparable image quality with the ones from a separated reconstruction using recorded physiological signal based on the same data (c). SG-MUSIC has shorter scan time yet better image quality (c,d) than the original MUSIC (b). Detailed coronary vessel structures are visualized in the reformatted SG-MUSIC images(e). (RESP: airway pressure signal; RSG: respiratory SG; CSG: cardiac SG; TA: acquisition time).

The time series of SG projections show that the inward quasi-spiral significantly reduces the eddy-current artifacts on SG signals. The k-space pattern shows the segmented golden ratio provides are much uniform k-space sampling after data sorting (Fig. 1). In-vivo results in Fig. 2 show the SG signal corresponds well with the recorded physiological signal and SG images is comparable with the ones using recorded physiological signal on the same data. The SG-MUSIC (TA = 6 min) has better image quality than original MUSIC (TA = 8 min) due to the use of ROCK sampling pattern and ESPIRiT reconstruction. The volumetric images allows reformatting into arbitrary planes for visualization of detailed vascular structures.

## Conclusions

The proposed SG-MUSIC eliminates the need of physiological signal for motion gating, has slightly increased scan efficiency, while exceeding the image quality of the original MUSIC.

